# Development of an Effective Double Antigen Sandwich ELISA Based on p30 Protein to Detect Antibodies against African Swine Fever Virus

**DOI:** 10.3390/v14102170

**Published:** 2022-09-30

**Authors:** Mengxiang Wang, Jinxing Song, Junru Sun, Yongkun Du, Xiaodong Qin, Lu Xia, Yanan Wu, Gaiping Zhang

**Affiliations:** 1 College of Veterinary Medicine, Henan Agricultural University, Zhengzhou 450046, China; 2 International Joint Research Center of National Animal Immunology, Zhengzhou 450046, China; 3 Longhu Laboratory, Zhengzhou 450046, China; 4 Henan Engineering Laboratory of Animal Biological Products, Zhengzhou 450046, China

**Keywords:** African swine fever virus, double-antigen sandwich ELISA, diagnosis, p30 protein, prokaryotic expression system

## Abstract

African swine fever (ASF), the highly lethal swine infectious disease caused by the African swine fever virus (ASFV), is a great threat to the swine industry. There is no effective vaccine or diagnostic method to prevent and control this disease currently. The p30 protein of ASFV is an important target for serological diagnosis, expressed in the early stage of viral replication and has high immunogenicity and sequence conservatism. Here, the CP204L gene was cloned into the expression vector pET-30a (+), and the soluble p30 protein was successfully expressed in the *E. coli* prokaryotic expression system and then labeled with horseradish peroxidase (HRP) to be the enzyme-labeled antigen. Using the purified recombinant p30 protein, a double-antigen sandwich ELISA for ASFV antibody detection was developed. This method exhibits excellent specificity, sensitivity and reproducibility in clinical sample detection with lower cost and shorter production cycles. Taken together, this study provides technical support for antibody detection for ASFV.

## 1. Introduction

African swine fever (ASF) is an acute, hemorrhagic, viral infectious disease caused by the African swine fever virus (ASFV), which can infect domestic pigs and various wild boars, mainly transmitted by soft ticks [1]. The clinical symptoms of pigs infected with ASFV are fever (40–42 °C), rapid heartbeat, respiratory distress, cyanosis of skin, obvious bleeding of lymph nodes, kidney and gastrointestinal mucosa, and the mortality rate after infection in domestic pigs is close to 100% [2]. Since being first diagnosed in Kenya in the 1920s, ASF has gradually spread to various regions of the world. The outbreak of ASF in China has resulted in hundreds of millions of pigs dying or being slaughtered since 2018 because no vaccines or drugs are available [3]. Thus, establishing an early diagnosis method can effectively block ASF spread and minimize losses [4]. Strengthening basic research is the key to ASF prevention and control [5].

ASFV is a double-stranded Nucleocytoplasmic Large DNA Virus (NCLDV) and is the only species under the family of African swine fever viruses [6]. The ASFV particle has an icosahedral morphology structure with a capsule and capsid and can reach 175–215 mm in diameter [7]. ASFV encodes at least 150 proteins during viral replication; more than fifty of which are eventually packaged into viral particles and play major functions in viral infection. The p30 protein is expressed early and involved in the internalization of the virus after its adsorption to the host cell. It is encoded by the CP204L gene, has a relative molecular mass of 30 kDa, and is localized in the cytoplasm of infected cells [8,9]. The p30 protein is also one of the most antigenic proteins in ASFV, triggering the production of neutralizing antibodies in infected animals [10,11]. Therefore, the p30 protein is a key antigenic protein for serological diagnosis and an important target for ASFV antibody detection.

**Figure 1:**
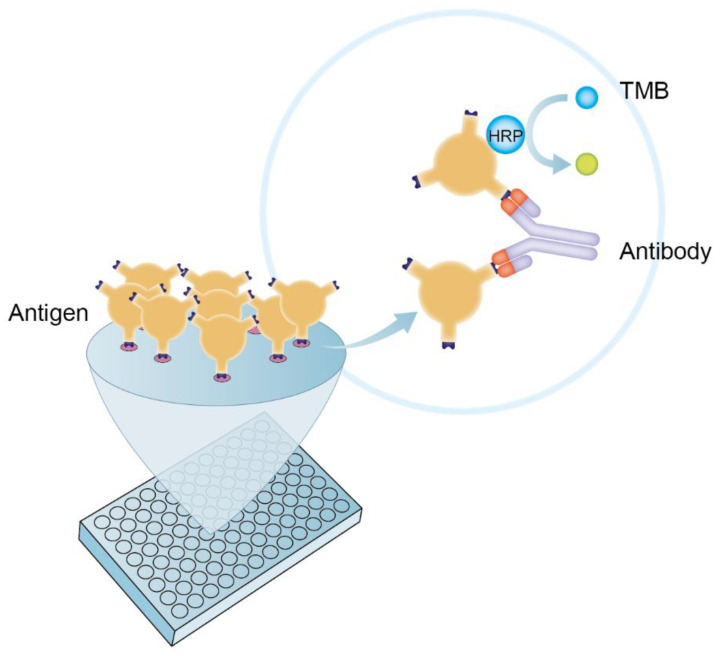
Schematic diagram of DAgS-ELISA Principle. TMB: 3,3,5,5-tetramethylbenzidine; HRP: horseradish peroxidase.

Enzyme-linked immunosorbent assay (ELISA) is an analytical technique for detecting the presence of an antigen or antibody in a specific sample. It is widely applied in clinical diagnosis, pathological studies, and quality control studies and has the advantages of simple operation, high sensitivity, and specificity. ELISA has been developed into various types according to different serological principles, including direct ELISA, indirect ELISA, sandwich ELISA, competitive ELISA, multiplex ELISA, and so on. The double-antigen sandwich ELISA (DAgS-ELISA) involved here uses a specific enzyme-conjugated antigen to detect the corresponding antibody (Figure 1). This method can effectively reduce the occurrence of false positives and has higher sensitivity and specificity compared with other methods [12,13]. In recent years, syphilis [14], HIV [15], hepatitis c [16], and other research fields have successfully used the double-antigen sandwich method to achieve the accurate detection of the corresponding antibodies. In this study, a highly sensitive and specific double-antigen sandwich ELISA method for p30 was established to provide technical support for the detection of the ASFV antibody and facilitate the prevention of ASF.

## 2. Materials and Methods

### 2.1. Serum Samples

Positive standard sera for ASFV, pseudorabies virus (PRV), classical swine fever virus (CSFV), and porcine circovirus type 2 (PCV2) were purchased from China Veterinary Culture Collection Center (CVCC, Beijing, China). Positive serum samples for porcine reproductive and respiratory syndrome virus (PRRSV), porcine parvovirus (PPV), foot-and-mouth disease virus (FMDV), negative serum samples for ASFV, and clinical serum samples were collected previously and stored in the laboratory. 

### 2.2. Expression and Identification of p30 Protein

**Figure 2:**
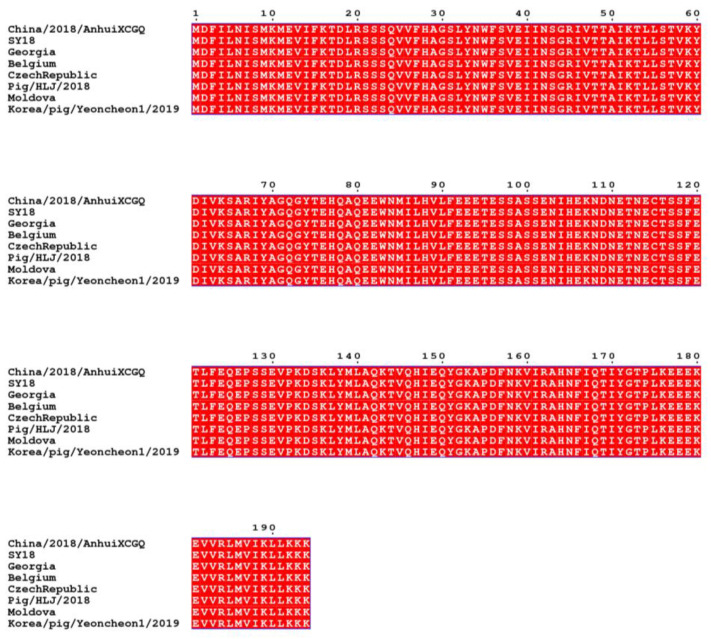
Comparison of CP204L sequences of different virulent strains.

The full-length CP204L coding region of ASFV (GenBank accession, No. MK128995.1) was synthesized by Tsingke Biotechnology Co., Ltd. (Zhengzhou, China) and amplified using F (agcttgtcgacggagctcgaattcttattttttttttaa) and R (caaggccatggctgatatcggatccatggattttatttta) primers appended with *Eco*RI and *Bam*HI restriction sites, respectively. The CP204L gene sequence in this strain did not differ from the ASFV-SY18 strain of the first outbreak of African swine fever in Shenyang, Liaoning Province, China, and was completely identical to the CP204L sequences of some other major prevalent strains after comparison (Figure 2), so the CP204L sequence of this strain was selected to be sufficient to detect various domestic popular strains [17]. The target fragment was subcloned into the pET-30a (+) vector using the homologous recombination technique. The recombinant plasmid was transformed into *E. coli* DH5α competent cells and was verified by double restriction enzyme digestion. The recombinant plasmid was transformed into *E. coli* BL21 competent cells and protein expression were induced with 0.2 mM IPTG at 16 °C for 14 h. The proteins were purified using Nickel–nitrilotriacetic acid (Ni-NTA) metal affinity chromatography and identified by SDS-PAGE and Western blot. ASFV-positive serum was used as the primary antibody (dilution of 1:1000) in a Western blot assay. The HRP-conjugated p30 protein was prepared using the SureLINKTM HRP Conjugation Kit (KPL, Gaithersburg, MD, USA) according to the instructions. Activation of the sugar hydroxyl groups on HRP by sodium periodate (NaPIO_4_) generates aldehyde groups that can couple with the primary amines in the p30 protein, immediately followed by a reduction in Schiff bases and the formation of stable conjugates.

### 2.3. The Establishment of DAgS-ELISA Based on p30 Protein

The square matrix titration is used to screen the optimal coating concentration of antigen and the optimal dilution of negative-positive serum. The p30 protein was coated at a concentration of 0.5–8 µg/mL, while positive and negative standard sera were incubated at a dilution of 1:5–1:80. We used carbonate buffer (CBS, 0.05 mol/L, pH = 9.6), NaHCO_3_ (0.05 mol/L, pH = 9.6), and phosphate buffer (PBS, pH = 7.3) coated with antigen under the tested coating conditions (37 °C for 1 h, 37 °C for 2 h, 4 °C for 6 h, or 4 °C overnight). The optimal blocking solution was selected from 3%, 5%, 8% skimmed milk and 3%, 5%, 8% Bo-vine serum albumin, and then blocked at 37 °C for 1 h, 37 °C for 2 h, 4 °C for 6 h, or directly blocked overnight at 4 °C. The sera were incubated for 0.5, 1, 1.5, 2, and 2.5 h, respectively, to select the optimal incubation time. The HRP-labeled p30 protein was diluted in the ratio of 1:500, 1:1000, 1:2000, 1:4000, and 1:6000, respectively, and reacted for 0.25, 0.5, 1, 1.5, and 2 h, respectively, to obtain the optimal conditions for HRP-labeled p30 protein. After three rounds of washing, 100 µL of 3,3,5,5-tetramethylbenzidine (TMB) substrate solution was added and incubated at room temperature (RT) for 15 min. The reaction was stopped by the addition of 50 µL of 2 M sulfuric acid, and the absorbance was measured on a microplate spectrophotometer at a wavelength of 450 nm. All samples are tested in duplicate.

### 2.4. Determination of the Cut-Off Value

Fifty negative clinical sera were used to calculate the threshold values for the DAgS-ELISA. The mean value ($\bar{X}$) and standard deviation (SD) of the OD450nm were calculated by statistical analysis. The cut-off value was determined as$\;\bar{X}$ + 3SD. When the OD450nm value of the sample was greater than or equal to the cut-off, it was determined to be positive. If not, the results should be judged as negative.

### 2.5. Assessment of the Diagnostic Sensitivity and Specificity

The established DagS-ELISA was used to detect CSFV, PRRSV, PCV2, PRV, FMDV, ASFV-positive, and ASFV-negative sera. ASFV-positive sera were diluted from 1:10 to 1:2560, and the DagS-ELISA assay was performed according to the optimized conditions, the OD450nm value was measured, and the change in the value with the increase in serum dilution was observed.

### 2.6. Reproducibility of DagS-ELISA

The intra- and inter-batch reproducibility of the established DagS-ELISA was determined using 10 sera with known backgrounds (five negative and five positive). The OD450nm values of each serum were read repeatedly five times.

### 2.7. Comparison of DagS-ELISA with Commercial Kits

All the clinical serum samples were detected with both the African swine fever virus block ELISA Antibody Test Kit (Qingdao Lijian Bio-Tech Co., Ltd., Qingdao, China) and the established DAgS-ELISA, and then the results were analyzed and compared.

### 2.8. Statistical Analysis

All data were visualized using the GraphPad Prism 8.0.2 software (GraphPad Software, San Diego, CA, USA).

## 3. Results

### 3.1. Expression and Purification of p30 Protein

**Figure 3:**
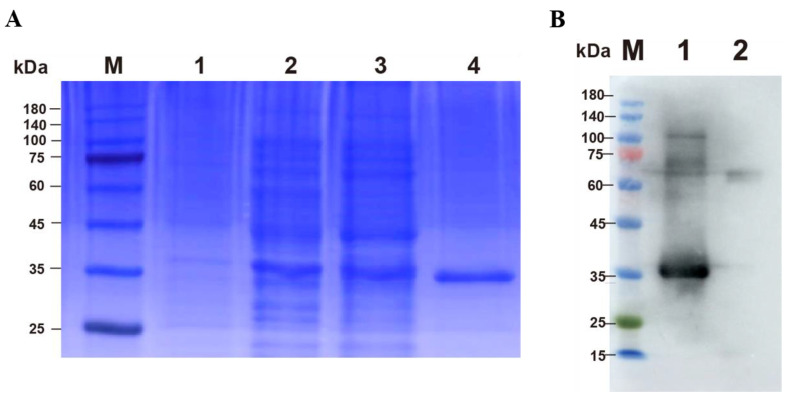
Results of p30 expression and purification. (**A**) Expression and purification of p30 protein. Lane M: protein marker (25 kDa–180 kDa); Lane 1: uninduced *E. coli* BL21 culture; Lane 2: induced *E. coli* BL21 lysate; Lane 3: soluble fraction; Lane 4: the purified p30 protein. (**B**) Western blot analysis of purified p30 protein. ASFV-positive serum was used as the primary antibody (dilution of 1:1000) here. Lane M: protein marker (10 kDa–180 kDa); Lane 1: p30 protein; Lane 2: Non-induced *E coli*. Lysate.

The p30 protein was successfully expressed in the soluble fraction and confirmed by Western blot using ASFV-positive pig serum (Figure 3).

### 3.2. Standardization of the DAgS-ELISA Procedure

By checkerboard titration tests, the OD value gave the maximum difference be-tween the positive serum and negative serum (P/N value of 13.316) when the dilutions of antigen and serum were 1 μg/mL and 1:10, respectively (Table 1). Therefore, the final concentration of coating antigen was 100 ng/well by calculation, and the optimal dilution of the serum was 1:10, respectively. At the same time, the reaction temperature, time, and other conditions were optimized by the index of the P/N value.

**Figure 4:**
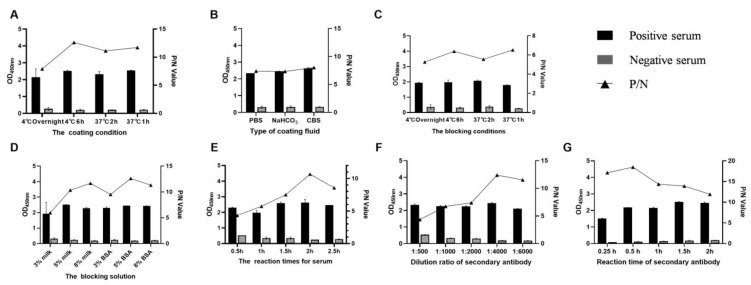
Optimization results for the DAgS-ELISA procedure. (**A**): Determination of optimal coating conditions; (**B**): Determination of the best coating solution; (**C**): Determination of optimal blocking conditions; (**D**): Determination of the best blocking solution; (**E**): Optimal incubation time for serum; (**F**): Optimal dilution of HRP-labeled p30 protein; (**G**): Optimal incubation time for HRP-labeled p30 protein.

First, the optimal coating conditions were screened from different coating temperatures and times, including 4 °C overnight, 4 °C for 6 h, 37 °C for 2 h, and 37 °C for 1 h, and the final optimal combination was 4 °C for 6 h with the maximum P/N (Figure 4A). Then, under the above optimal coating conditions, the type of coating solutions was optimized, and according to Figure 4B, the best coating solution was CBS. By analogy, the optimal blocking condition, blocking solution, reaction times for serum, dilution ratio of enzyme-labeled antigen, and reaction time of enzyme-labeled antigen were optimized sequentially under the determined optimal conditions. Finally, the optimal combinations for the DAgS-ELISA were coating the plate with p30 protein in CBS for 6 h at 4 °C, followed by blocking with 5% BSA for 1 h. The optimal incubation time for serum is 2 h, while the optimal incubation time for enzyme-conjugated antigen is 30 min at a 1:4000 dilution (Figure 4).

### 3.3. Cut-Off Value of the DAgS-ELISA

**Figure 5:**
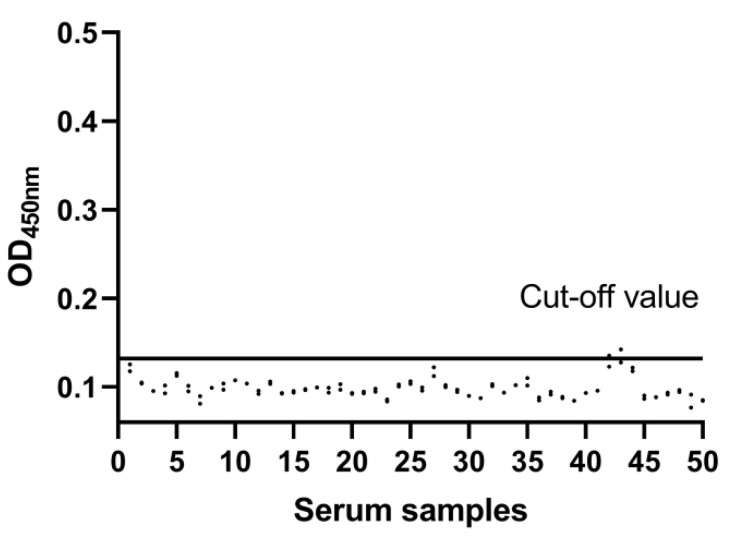
Determination of the cut-off value of the DAgS-ELISA (OD450nm = 0.132).

The optimized DAgS-ELISA conditions were used to detect 50 negative sera. The average ($\bar{X}$) of 50 negative sera coated with p30 protein was 0.098 and the standard deviation (SD) was 0.011, resulting in a cut-off value of ($\bar{X}$ + 3SD) = 0.132 (Figure 5). The serum samples with OD450nm values ≥ cut-off value were determined to be positive. If not, the serum should be considered negative.

### 3.4. Diagnostic Sensitivity and Specificity of DAgS-ELISA

**Figure 6:**
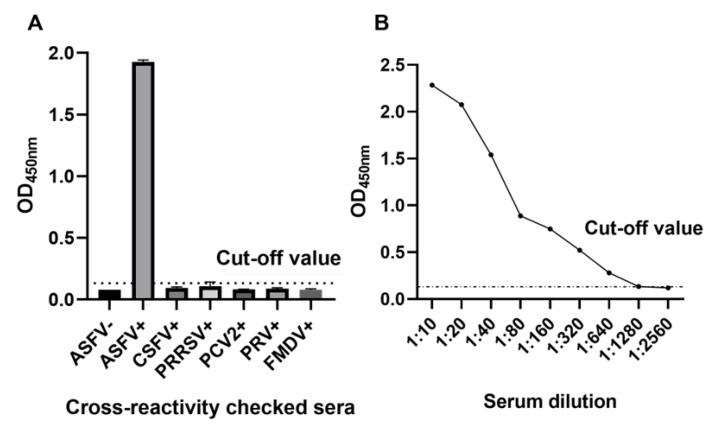
Sensitivity and specificity of the DAgS-ELISA. (**A**): Specificity test of the DAgS-ELISA. The DAgS-ELISA detected no cross-reactions with sera containing antibodies against five other porcine pathogens, including CSFV, PRRSV, PCV2, PRV, and FMDV; (**B**): Sensitivity of the DAgS-ELISA.

ASFV standard sera were detected using the DAgS-ELISA with optimized conditions, and the OD450nm values of positive sera were higher than the cut-off value, while the negative sera and other common swine disease sera were lower than the cut-off value (Figure 6A), indicating the good specificity of the established DAgS-ELISA. The sensitivity of the DAgS-ELISA method established is 1:1280 (Figure 6B).

### 3.5. Repeatability of the DAgS-ELISA

Intra- and inter-batch variation assays were used to evaluate the reproducibility of DAgS-ELISA. The coefficient of variation (CV) is the ratio of the standard deviation to the mean and reflects the degree of data dispersion. The lower the CV, the better the reproducibility of the method. According to the results in Table 2, the intra-batch CVs of the samples were all below 5%, and the inter-batch CVs were all below 10%, indicating that the DAgS-ELISA has a high degree of reproducibility.

### 3.6. Clinical Serum Sample Detection

**Figure 7:**
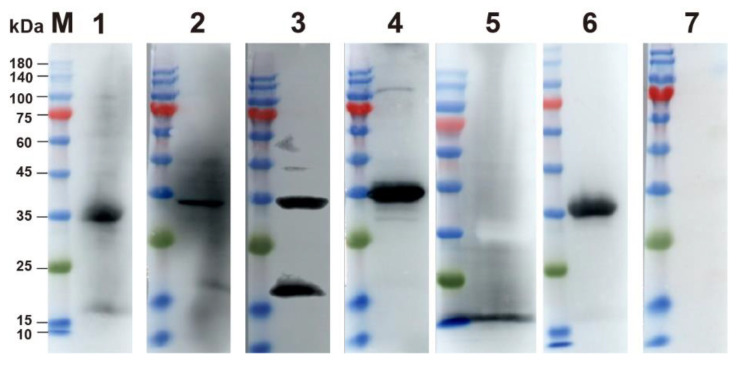
Western Blot results of five clinical sera. Line M: protein markers (10 kDa–180 kDa); lines 1–5: five clinical sera with controversial. The results show that serums 1–4 are positive, while 5 is negative; line 6: Standard positive serum; line 7: Standard negative serum.

The results of clinical serum sample detection using established DAgS-ELISA and the commercial kits are shown in Table 3. Five of the same serum samples tested positive by DAgS-ELISA but negative by the commercial kit. For further confirmation, a western blot was performed, which finally showed four positive and one negative (Figure 7). It can be concluded that the DAgS-ELISA established here has high accuracy.

## 4. Discussion

African swine fever has been widespread in several countries in Africa, Asia, and Europe since its first appearance in 1921. On 3 August 2018, China confirmed the first outbreak of African swine fever by the Ministry of Agriculture and Rural Affairs (MARA). Since then, ASF has spread to almost all the provinces in less than two years, causing huge economic losses [18]. However, there is no vaccine or other effective treatment for this disease [19,20]. Therefore, having an accurate diagnostic method becomes the key point in the prevention and control of ASF and the restoration of the pig industry. At present, commonly used detection methods mainly include ELISA, Western blot, indirect immunofluorescence (IIF), indirect immune-peroxidase test (IPT), etc. [21]. Among these methods, ELISA serological diagnosis technology is the most mature and stable one, and simple to operate. In addition, it can perform batch testing of samples, which is suitable for wide application in clinical practice [22].

Currently, many different diagnostic methods for ASF have been established based on different principles and operations. For example, Kexin Zhong et al. established an indirect ELISA method based on the pp62 protein of ASFV [23], and Xuexiang Yu et al. established a blocking ELISA method based on monoclonal antibodies against p30 [24]. However, the secondary antibody in the indirect ELISA method mainly recognizes the Fc region of the primary antibody and, therefore, only specific types of antibodies can be detected, while some non-specific antibodies in the competing ELISA method bind to the encapsulated protein and thus affect the accuracy of the results. The DAgS-ELISA established here is based on the simultaneous binding of specific antigens to the Fab regions on both sides of the antibody, so it can not only detect various types of antibodies in the sample, including IgM and IgG but also avoid the interference of non-specific antibodies. Moreover, DAgS-ELISA has higher sensitivity and specificity [25]. Furthermore, the current global epidemic of coronavirus disease 2019 (COVID-19) has selected DAgS-ELISA among many ELISA methods, reflecting the application potential of this method in pandemics [26,27]. To our knowledge, this method is the first attempt to be applied to the detection of ASF.

At present, commercial kits on the market have been widely used in clinical sample testing, such as ID. Vet’s competition ELISA kit, Ingenasa’s competition ELISA kit, etc. [2]. In ELISA methods, the secondary antibodies or blocking antibodies are time-consuming and laborious. While in this study, neither secondary antibodies nor blocking antibodies are required. Soluble p30 protein with good immunogenicity and reactogenicity was expressed in a short time using the prokaryotic expression system, which can be easily labeled by HRP. Therefore, the DAgS-ELISA established here will have a huge potential advantage in terms of the production cycle, price, etc., and will provide technical support for the rapid and effective detection of ASF, as well as a reference for the detection of other infectious diseases.

The structural proteins p72, p54, and p30 of ASFV have high immunogenicity and conservation, usually as the main targets for serological diagnosis; notably, the p30 encoded by the CP204L gene has the highest expression in the early stage of viral replication and is the most immunogenic protein [28]. In addition, antibodies with a neutralizing effect on the p30 protein have been reported to be detectable on day 8 after viral infection, and the ELISA detection method using p30 as the coating antigen can basically be used for the whole process of monitoring after ASFV infection [29]. Cubillos et al. simultaneously evaluated the reactogenicity of recombinant p30, p54 and p72 proteins in a single reaction and demonstrated that p30 could serve as the optimal diagnostic antigen [21]. In this study, the soluble p30 protein was successfully expressed and validated antigenicity, and a double-antigen sandwich ELISA was further established on this basis.

In summary, specificity studies have demonstrated that the DAgS-ELISA method established here does not cross-react with antibodies against other swine disease-related viruses, such as PRRSV, PCV2, PRV, FMDV, and CSFV. Sensitivity studies have shown that this method can detect ASFV-positive sera at a maximum dilution of 1:1280. The results of the repeatability test showed that the CV of the intra-assay repeatability test was <5%, and the CV of the inter-assay repeatability test was less than 10%, indicating good repeatability. This DAgS-ELISA for ASFV antibody detection shows excellent performance compared with commercial kits and can be used for clinical serum sample detection. At the same time, considering the low cost and short production cycle of the detection method, it will lay an important foundation for the further development of ASF antibody detection kits, which is of great significance for the prevention, control, and eradication of ASF.

## Figures and Tables

**Table 1:** Determination of optimal antigen coating concentration and serum dilutions.

Dilution of Sera		Antigen at Different Concentrations (μg/mL)					
		0.5	1	2	4	6	8
1:5	P	2.635	2.7043	2.593	1.751	1.264	1.433
	N	0.333	0.2111	0.222	0.238	0.256	0.267
	P/N	7.917	12.811	11.693	7.349	4.938	5.357
1:10	P	2.430	2.560	2.437	0.904	0.528	0.479
	N	0.252	0.192	0.218	0.224	0.237	0.252
	P/N	9.638	13.316	11.192	4.027	2.225	1.903
1:20	P	2.235	2.247	1.836	1.836	0.264	0.406
	N	0.261	0.194	0.187	0.187	0.262	0.284
	P/N	8.579	11.572	9.831	1.760	1.007	1.427
1:40	P	1.656	1.616	0.813	0.309	0.256	0.255
	N	0.3046	0.160	0.172	0.201	0.225	0.239
	P/N	5.435	10.131	4.736	1.540	1.137	1.067
1:80	P	0.971	0.739	0.446	0.322	0.267	0.296
	N	0.314	0.167	0.179	0.223	0.198	0.259
	P/N	3.089	4.414	2.500	1.448	1.348	1.143

Notes. The black bold value indicates the value under the optimal condition chosen for subsequent DAgS-ELISA. P: OD value of positive samples; N: OD value of negative samples.

**Table 2:** Results of the repeatability assay for DAgS-ELISA.

Sample No.		Intra-assay CV (%)		Inter-assay CV (%)	
		X ± SD	CV%	X ± SD	CV%
Positive samples	1	1.687 ± 0.049	2.88	1.680 ± 0.063	3.74
	2	1.645 ± 0.021	1.28	1.534 ± 0.049	3.22
	3	1.667 ± 0.052	3.15	1.668 ± 0.105	6.32
	4	1.682 ± 0.057	3.37	1.753 ± 0.115	6.54
	5	1.893 ± 0.033	1.76	2.028 ± 0.193	9.50
Negative samples	6	0.099 ± 0.002	1.62	0.086 ± 0.002	2.70
	7	0.087 ± 0.003	3.73	0.070 ± 0.005	7.38
	8	0.090 ± 0.002	2.74	0.086 ± 0.008	9.51
	9	0.079 ± 0.003	3.63	0.077 ± 0.002	2.38
	10	0.085 ± 0.003	3.85	0.084 ± 0.004	5.10

**Table 3:** Comparison results of DAgS-ELISA and commercial kits.

No. of Clinical Samples	DAgS-ELISA		Commercial Kits	
	No. of Positive	Positive Rate (%)	No. of Positive	Positive Rate (%)
120	26	21.7%	21	17.5%

## Data Availability

The data analyzed during the current study are available from the corresponding author upon reasonable request.
